# Anti-Inflammatory Properties of Injectable Betamethasone-Loaded Tyramine-Modified Gellan Gum/Silk Fibroin Hydrogels

**DOI:** 10.3390/biom10101456

**Published:** 2020-10-17

**Authors:** Isabel Matos Oliveira, Cristiana Gonçalves, Myeong Eun Shin, Sumi Lee, Rui Luis Reis, Gilson Khang, Joaquim Miguel Oliveira

**Affiliations:** 1 3B’s Research Group, I3Bs—Research Institute on Biomaterials, Biodegradables and Biomimetics of University of Minho, Headquarters of the European Institute of Excellence on Tissue Engineering and Regenerative Medicine, Avepark, Parque de Ciência e Tecnologia, Zona Industrial da Gandra, Barco, 4805-017 Guimarães, Portugal; isabel.oliveira@i3bs.uminho.pt (I.M.O.); cristiana.goncalves@i3bs.uminho.pt (C.G.); rgreis@i3bs.uminho.pt (R.L.R.); 2 ICVS/3B’s—PT Government Associate Laboratory, Braga, 4805-017 Guimarães, Portugal; 3 Department of BIN Fusion Technology, Department of Polymer Nanoscience and Polymer BIN Research Centre, Chonbuk National University, 567 Baekje-daero, Deokjin-gu, Jeonju 561-756, Korea; meshin@jbnu.ac.kr (M.E.S.); sumilee@jbnu.ac.kr (S.L.); gskhang@jbnu.ac.kr (G.K.)

**Keywords:** Ty–GG/SF hydrogels, enzymatic crosslinking, betamethasone, inflammation, rheumatoid arthritis

## Abstract

Rheumatoid arthritis is a rheumatic disease for which a healing treatment does not presently exist. Silk fibroin has been extensively studied for use in drug delivery systems due to its uniqueness, versatility and strong clinical track record in medicine. However, in general, natural polymeric materials are not mechanically stable enough, and have high rates of biodegradation. Thus, synthetic materials such as gellan gum can be used to produce composite structures with biological signals to promote tissue-specific interactions while providing the desired mechanical properties. In this work, we aimed to produce hydrogels of tyramine-modified gellan gum with silk fibroin (Ty–GG/SF) via horseradish peroxidase (HRP), with encapsulated betamethasone, to improve the biocompatibility and mechanical properties, and further increase therapeutic efficacy to treat rheumatoid arthritis (RA). The Ty–GG/SF hydrogels presented a β-sheet secondary structure, with gelation time around 2–5 min, good resistance to enzymatic degradation, a suitable injectability profile, viscoelastic capacity with a significant solid component and a betamethasone-controlled release profile over time. In vitro studies showed that Ty–GG/SF hydrogels did not produce a deleterious effect on cellular metabolic activity, morphology or proliferation. Furthermore, Ty–GG/SF hydrogels with encapsulated betamethasone revealed greater therapeutic efficacy than the drug applied alone. Therefore, this strategy can provide an improvement in therapeutic efficacy when compared to the traditional use of drugs for the treatment of rheumatoid arthritis.

## 1. Introduction

Among the several types of chronic inflammatory disorders, rheumatoid arthritis (RA) is a debilitating condition with severe physical, emotional and socioeconomic burdens. This disease affects 1% of the adult population worldwide, and there are approximately 5–50 new cases per 100,000 adults per year. The prevalence increases with age and occurs four times more often in women than men [1]. The disease starts with an inflammatory process that is characterized by inflammation in the synovial membrane, induced by leucocyte infiltration; joint swelling and pain; and at the same time, systemic inflammation registered by elevated serum levels of autoantibodies and acute phase protein; and later on, cartilage and bone tissue destruction [2]. The most common treatments for rheumatoid arthritis comprise nonsteroidal anti-inflammatory drugs, corticosteroids, disease-modifying antirheumatic drugs and some biological agents [3]. These drugs have improved quality of life for a significant number of RA patients. Corticosteroids, such as betamethasone, are inflammation suppressors that have been mainly used for the treatment of RA [4]. However, there are some concerns regarding the long-term use of corticosteroids because of the high doses, and consequently, the side effects. Therefore, it is necessary to develop and test new approaches to specifically target inflamed joints and attenuate damage to healthy tissues [5,6].

Injectable hydrogels have been widely studied, particularly in the tissue engineering and regenerative medicine field. These promising biomaterials present several advantages, such as convenient injectability with minimal invasiveness, and the ability to fill irregular and defective areas entirely and produce three-dimensional, intensely hydrated structures [7]. Furthermore, the injectable hydrogels can also be suitable for non-invasive applications; they are applied in the local delivery of a wide range of therapeutic agents, including biomolecules and drugs. Injectable hydrogels derived from natural polymers, such as silk fibroin, alginate, hyaluronic acid and collagen, have been regularly used and show great potential for biomedical applications, including the delivery of local therapeutic agents [8]. The promising advantages of these biomaterials come from their adaptability and versatility due to their properties and components being similar to those of the physiological extracellular matrix [9].

Natural polymers are often used as hydrogels for controlled drug delivery vehicles. The hydrogels can be prepared with natural and controlled compositions and morphologies. The properties can be influenced to enhance biocompatibility, immune compatibility, cellular uptake, stability and solubility [10]. Silk is a natural fiber produced by silkworm (*Bombyx mori*) cocoons, and it is composed of two types of protein (fibroin and sericin). Silk fibroin has been extensively studied as a drug delivery system for its unique and highly versatile structure and its strong clinical track record. Silk hydrogels have unlimited applications and have several advantages, such as high biocompatibility, tunable biodegradation and low immunogenicity [11,12]. Silk fibroin solution has been used to produce hydrogels as injectable systems for load-bearing of cartilage tissue [13]. Most silk fibroin hydrogels are formed in a sol–gel transition, with a conformation transition from the amorphous structure to β-sheet, through different physical and chemical procedures. Enzymatically crosslinking methods have been used to produce in situ hydrogels [14,15]. Compared to other systems, enzyme-mediated crosslinking can present several advantages; for instance, gelation time can be easily adjusted to be fast, favoring drug delivery. The enzymatic crosslinking via horseradish peroxidase (HRP) has shown great potential once the phenol groups in tyrosine, tyramine and/or aminophenol can be conjugated in the presence of hydrogen peroxide (H_2_O_2_) [16,17]. Since the silk protein contains tyrosine amino acids, this natural polymer has great advantages for producing SF hydrogels [18].

Nevertheless, usually, natural polymeric materials are not mechanically stable enough, and the rate of biodegradation is high [19]. Thus, to increase mechanical stability, natural or synthetic materials can be used to produce composite structures with biological cues to promote tissue-specific interactions [20].

Gellan gum is a water-soluble extracellular polysaccharide produced by the aerobic fermentation process of *Sphingomonas elodea*. According to the degree of acyl substitution, it is available in two forms, high acyl and low acyl, the low acyl form being the most common and commercially available one [21]. The GG has properties that provide great versatility, such as biocompatibility, biodegradability, easy bio-fabrication, tunable mechanical properties, cell adhesion and easy functionalization. These appealing properties make GG a promising material in different tissue engineering fields, such as drug delivery [21,22].

Previously, gellan gum was successfully modified with tyramine (Ty–GG), to produce hydrogels enzymatically crosslinked by HRP [23]. The Ty–GG hydrogels showed good strength and resistance, did not interfere with metabolic activity and cell proliferation, and presented good therapeutic efficiency; i.e., Ty–GG hydrogels with a corticosteroid encapsulated showed a great decrease in inflammation as compared to the administration of the corticosteroid alone. Therefore, the Ty–GG was used to increase the mechanical stability of silk fibroin and further enhance the therapeutic effect as a drug delivery system.

In this work, we developed Ty–GG/SF (silk fibroin) hydrogels with encapsulated betamethasone to be injected into intra-articular cavities to further increase therapeutic efficacy in the inflamed joints of patients with RA.

## 2. Materials and Methods

### 2.1. Physicochemical Characterization

#### 2.1.1. Preparation of Silk Fibroin Solution

The silk fibroin solution was obtained based on a previously reported procedure [24,25].

Silkworm cocoons (Rural Development Administration, Jeonju, Korea and Portuguese Association of Parents and Friends of Mentally Disabled Citizens (APPACDM, Castelo Branco, Portugal)) were cut into fragments and boiled in 0.02 M Sodium carbonate (Na_2_CO_3_) solution (Showa Chemical, Tokyo, Japan) for 30 min to remove sericin. Boiled silkworm cocoons were washed with distilled water three times and dried inside an oven at 70 °C. The dry silkworm cocoons were then dissolved in the oven with 9.3 M LiBr (lithium bromide; Kanto Chemical, Tokyo, Japan) at 70 °C for 4 h. The dissolved solution was dialyzed using a SnakeSkin Dialysis Tubing 3500 MWCO (ThermoScientific, Waltham, MA, USA), over 48 h, against distilled water, to remove LiBr. Silk fibroin was kept at 4 °C until use.

#### 2.1.2. Horseradish Peroxidase to Induce Ty–GG/SF Hydrogel Formation

Ty–GG previously produced by the authors was used to obtain Ty–GG/SF hydrogels [23]. Briefly, gellan gum from Gelzan™ CM (Sigma-Aldrich, St. Louis, MO, USA) was dissolved in water to a final concentration of 1% (*w*/*v*) and sodium (meta)periodate (Sigma-Aldrich, St. Louis, MO, USA) was added to a final concentration of 1 mM in GG at 1% (*w*/*v*). The reaction was stopped by adding glycerol (500 mM) and oxidized GG was precipitated from the reaction mixture by adding NaCl at 1% (*w*/*v*) and 2 volumes of 99% (*v*/*v*) ethanol. The precipitate was separated, dried and dissolved in 0.1 M sodium phosphate buffer, pH 6, and tyramine hydrochloride (Sigma-Aldrich, St. Louis, MO, USA) was added through molecular equivalents (number of mol equivalents of GG to number of mol equivalents of tyramine). The solid cyanoborohydride was added at 0.5% (*w*/*v*) final concentration and modified gellan gum was precipitated by adding NaCl to 1 M final concentration and two volumes of 96% (*v*/*v*) ethanol. Ty–GG was obtained after dialysis for 7 days and lyophilized for approximately 4 days.

Horseradish peroxidase (HRP) solution (0.84 mg mL^−1^) (Sigma-Aldrich, St. Louis, MO, USA) and hydrogen peroxide solution (H_2_O_2_, 0.36% (*v*/*v*)) (VWR, Radnor, PA, USA) were both prepared in water. Tyramine-gellan gum/SF hydrogels were prepared by mixing the 1% (*w*/*v*) Ty–GG and 2% (*w*/*v*) SF (1:1) solutions with different amounts of HRP and H_2_O_2_ solutions (Table 1). Ty–GG/SF hydrogels were prepared by adding 200 µL of the mixture solutions in a polypropylene mold at 37 °C.

#### 2.1.3. Analysis of the Secondary Protein Structure of Silk Fibroin

The secondary structure of the SF in the Ty–GG/SF hydrogels was recorded by Fourier transform infrared (FTIR) spectroscopy (IRPrestige 21, Shimadzu, Japan). Measurements were made with attenuated total reflection (ATR) over a Germanium crystal. Transmission spectra were acquired on an IR Prestige-21 spectrometer (Shimadzu, Japan), using 32 scans, a resolution of 4 cm^−1^ and a wavenumber range between 4400 and 400 cm^−1^. The SF absorbance spectra were calculated using Equation (1).

*Abs*(absorbance) = 2 − log_10_*T*(transmittance/%) (1)

To quantify secondary structure composition, Origin Pro 2018 Software was used. The deconvolution of the amine I region (1750–1600 cm^−1^) was performed using a Gaussian Peak shape and the bands were identified by the area integral (Area IntgP) analysis with the points of interest. Baseline treatment was performed on the deconvolution spectrum. A maximum entropy Gaussian curve fitting algorithm was executed on the deconvolution spectrum using the peak positions identified in the Area IntgP, maximum peak and variable peak height. Peak positions were attained using wavenumber ranges described by Litvinov et al. [26]. The structural composition was measured by relative areas of the fit curve in the amine I region.

#### 2.1.4. Water Uptake and Enzymatic Degradation of Ty–GG/SF Hydrogels

The water uptake ratio of the Ty–GG/SF hydrogels was measured in PBS solution at 37° C. The initial weight (w_i_) of each sample was measured, and then the hydrogels were immersed in PBS solutions. At the end of each time point (24, 72, 168, 336 and 504 h), the samples were collected and placed in filter paper to remove excess liquid, and the wet weight (w_w_) was measured. The percentage of water uptake was determined using Equation (2).

Water uptake (%) = (w_w_ − w_i_)/(w_i_) × 100 (2)

The durability of Ty–GG/SF hydrogels was evaluated by enzymatic degradation test. The enzyme, protease from *Streptomyces griseus* type XIV (Sigma-Aldrich, St. Louis, MO, USA) was prepared at 1 and 3.3 U mL^−1^ by dissolution in PBS. The initial weight of each sample was measured, and then the hydrogels were immersed in a protease solution. The dry weight (w_f_) was evaluated after samples were dried at 70 °C and until they reached a constant weight. The study was done at 37 °C at different time points, 3 replicates per time points, for 21 days. The weight loss was calculated using Equation (3).

Weight Loss (%) = (w_i_ − w_f_)/(w_i_) × 100 (3)

#### 2.1.5. Gelation Time, Injectability and Rheological Properties of the Ty–GG/SF Hydrogels

The gelation time was assessed after mixing Ty–GG/SF (1:1) with HRP and H_2_O_2_ in a vial at 37 °C in a water bath, which then was inverted to better analyze the hydrogel fixed in the bottom and measure how long hydrogel formation takes.

The injectability measurements were made utilizing a syringe with a 27 G needle in injectability equipment (PARALAB, Porto, Portugal). The syringe was filled with Ty–GG/SF that was then injected at a rate of 1 mL min^−1^ through the needle by applying the force exerted by the injectability machine. The injection force needed for each hydrogel condition was assessed, and water was used as control.

The mechanical properties of Ty–GG/SF hydrogels were assessed through an oscillatory mode in a rheometer (Kinexus pro+rheometer, software rSpace, from Malvern, Worcestershire, UK). The oscillatory experiments were performed using a plate–plate measuring system compound via an upper stainless-steel plate 8 mm in diameter, with a rough finish, so that the samples did not slip. The mechanical spectra (frequency sweep curves) were obtained from 0.1 to 10 Hz of frequency, at a shear strain of 0.3% for 5 min. All experiments were repeated 3 times at 37 °C. The shear strain used was within the linear viscoelastic region (LVER) of the material, previously obtained through strain sweep curves. By using a value from LVER it is ensured that the applied stress did not cause microstructural breakdowns (called yielding), and the hydrogel properties could be accurately determined.

#### 2.1.6. Betamethasone Release Studies of the Ty–GG/SF Hydrogels

To evaluate anti-inflammatory drug release systems, betamethasone was loaded in Ty–GG/SF hydrogels. The Ty–GG/SF hydrogels with encapsulated betamethasone (Sigma-Aldrich, St. Louis, MO, USA) were prepared by mixing Ty–GG/SF solution with 5 mg mL^−1^ of betamethasone in PBS, and the enzymatic crosslinking was obtained using the enzyme HRP and substrate H_2_O_2_. Each hydrogel was immersed in 1 mL of PBS to evaluate the betamethasone release profile. The samples were put in an incubator at 37°C and removed after 3, 6, 24, 72, 168, 336 and 504 h. The supernatant was removed and kept at −80° C until further analysis. The same hydrogels were used throughout the experiment.

A sequence of betamethasone dilutions was prepared (from 0 to 5 mg mL^−1^ in PBS) to obtain a calibration curve and assesses the amount of betamethasone released. The UV absorbance at 245 nm [27] was read in a microplate reader to measure the betamethasone release (EMax; Molecular Devices, Sunnyvale, CA, USA). Three samples per condition were evaluated at each time point.

### 2.2. In Vitro Studies

#### 2.2.1. Chondrogenic Cell Isolation

Firstly, after quarantine time for the New Zealand white rabbits, they were injected subcutaneously with a mixture of ketamine (15 mg/kg) and metedomidine (0.25 mg/kg) for anesthesia. The animals were euthanized by intravenous injection of an overdose of pentobarbital sodium (200 mg/kg). The knees from rabbits were removed, and the samples were washed 3 times with a PBS (1 X) solution. The cartilage was cut into small pieces and placed inside a conical tube with 1.5% (*v*/*v*) antibiotic–antimycotic. The sample was centrifugated at 300 G for 3 min at 4 °C, and the supernatant was discarded and replaced with a solution with DMEM/F12 medium and collagenase A (0.2 % in PBS) (Roche, USA), previously sterilized. The sample was kept in the CO_2_ incubator at 37 °C for 24 h. After incubation time, cells were centrifuged at 300 G for 3 min at 4 °C. The supernatant was removed, and cells were seeded on a flask with medium and kept in the CO_2_ incubator at 37 °C until they are ready to be used [28,29]. The institutional and national guidelines and certification for animal experimentation were followed.

#### 2.2.2. Cell Culture

In this work were used chondrogenic cells, isolated from female New Zealand white rabbits (Damul Sci., Jeonju, Korea) with 6 weeks old. The cells were cultured with Dulbecco’ s modified eagle medium (DMEM/F12) (Gibco, Waltham, MA, USA) and supplemented with 10% (*v*/*v*) fetal bovine serum (Gibco, Waltham, MA, USA) and 1% (*v*/*v*) antibiotic-antimycotic (Gibco, Seoul, South Korea) under standard conditions (37 °C in a humidified atmosphere containing 5% CO_2_).

The therapeutic effect was assessed in a human monocytic cell line (THP-1) (Sigma-Aldrich, St. Louis, MO, USA) (Sigma-Aldrich, St. Louis, MO, USA). RPMI 1640 Medium, GlutaMAX™ Supplement, HEPES (Thermo Fisher Scientific, Waltham, MA, USA) was used and supplemented with 10% (*v*/*v*) fetal bovine serum and 1% (*v*/*v*) of penicillin and streptomycin, under standard culture conditions (37 °C in a humidified atmosphere containing 5 vol% CO_2_).

#### 2.2.3. Metabolic Activity

The MTT assay was made to evaluate the metabolic activity of chondrogenic primary cells following MEM extract test.

The Ty–GG/SF hydrogels (C1, C2 and C3) were produced with a width ranging from 0.5 to 1 mm, with a minimum area of 60 cm^2^. The hydrogels were placed in a sterile tube with 10 mL of cell culture medium (DMEM-F12) for 24 h, in the water bath at 37 °C and 60 rpm. Simultaneously, 200 µL cell suspensions of chondrogenic primary cells were seeded into 96 well plates to achieve a cell density of 10,000 cells per cm^2^. After incubation time, the medium that was in contact with the Ty–GG/SF hydrogels (extraction fluid) was passed through a 0.45 µm membrane filter and added to chondrogenic primary cells. Cells with DMEM-F12 medium were used as controls. Test samples were incubated in triplicate. The cell culture medium was evaluated by 3-(4,5-dimethylthiazol-2-yl)-2,5-diphenyltetrazolium bromide (MTT, Sigma -Aldrich, Seoul, South Korea) at 24, 48 and 72 h. At each time point, the cell culture was refilled with a new culture medium, an MTT assay was done in a 9:1 ratio and the cell culture was incubated for 3 h. After violet crystals were produced, they were melted using dimethyl sulfoxide solution (DMSO), and 100 µL of solution from each well was transferred to 96-well plates. The optical density was read at 570 nm via microplate reader (EMax; Molecular Devices, Sunnyvale, CA, USA).

The MEM extract test was applied laid down in European and international standards (ISO10993-5).

#### 2.2.4. DNA Quantification

The total amount of DNA was assessed using a DNA quantification kit (Quant-iT™ PicoGreen^®^ dsDNA Assay Kit, Invitrogen, Waltham, MA, USA), to evaluate the effects of Ty–GG/SF hydrogels on chondrogenic primary cells. The protocol used for the seeding of cells and the handling of the Ty–GG/SF hydrogels was the same as that described in the previous section (MEM extract test). Briefly, extraction solution of Ty–GG/SF hydrogels (C1, C2 and C3) was incubated with chondrogenic cells at a density of 10,000 cells cm ^2^ for 24, 48 and 72 h. Cells cultured with DMEM-F12 medium were used as a positive control. After incubation time, the cells were washed with PBS solution and lysed with pure water. The cell solution was incubated for 1 h in a water bath at 37 °C and kept at −80 °C until further analysis. Several concentrations between 2 and 0 µL ml^−1^ were prepared to make the DNA calibration curve. The fluorescence was read using excitation of 480/20 nm and emission of 528/20 nm in a microplate reader, and DNA concentration was obtained from the standard curve.

#### 2.2.5. Fluorescence Microscopy

Chondrogenic primary cells were seeded using the same protocol and conditions described above. After that, cells were fixed with 10% (*v*/*v*) formalin (Sigma-Aldrich, St. Louis, MO, USA) and stained with 4,6-diamidino-2-phenylindole, dilactate (DAPI blue, VWR International, USA) for nuclei and Texas Red-X phalloidin (Sigma-Aldrich, St. Louis, MO, USA) for actin filaments of the cytoskeleton. Cells were observed through the fluorescence microscope (Axiolmager, Z1, Zeis Inc., Oberkochen, Germany).

#### 2.2.6. The Therapeutic Efficacy of Ty–GG/SF with Betamethasone Encapsulated

To assess anti-inflammatory activity, a THP-1 cell line stimulated with lipopolysaccharide (LPS) (Sigma-Aldrich, St. Louis, MO, USA) was used as an in vitro model. From the 3 conditions tested, the two that had the best results in metabolic activity and cell proliferation were evaluated (C1 and C3). THP-1 cells were seeded at a density of 1 × 10^6^ cells/mL in RPMI medium with 100 nM phorbol 12-myristate-13 acetate (PMA) (Sigma-Aldrich, St. Louis, MO, USA) for 24 h. After incubation time, the medium in the wells was refilled with RPMI medium without PMA and incubated for another 48 h. Cells were incubated with 100 ng mL^−1^ of LPS in RPMI medium and incubated for 5 h, to induce the inflammatory response. After this period, cells were cultured with the conditions previously mentioned (C1 and C3) for 7 days. At each time point, 1, 3 and 7 days, the culture medium was removed and stored at −80 °C for further analysis.

This test was done to evaluate the concentration of the cytokine TNFα in the medium after being in contact with the hydrogels. Human TNF-alpha DuoSET ELISA (R&D Systems, Minneapolis, MN, USA) kit and DuoSet Ancillary Reagent Kit 2 (R&D Systems, Minneapolis, MN, USA) were used. TNF-α standard solutions with concentrations from 1000 to 0 pg mL^−1^ were also evaluated in the ELISA plate to formulate the calibration curve. The optical density at 450 nm was read in a microplate reader (Synergy HT, BIO-TEK, Winooski, VT, USA).

### 2.3. Statistical Analysis

Statistical analysis was done with GraphPad Prism 8 version, where a Shapiro–Wilk normality test was formerly performed to evaluate the data normality. Statistical significance was obtained as * *p* < 0.05, ** *p* < 0.01 or *** *p* < 0.001. Results are presented as means ± standard deviations, and all assays were performed in triplicate.

## 3. Results and Discussion

### 3.1. Physicochemical Characterization

This study focused on the production of Ty–GG/SF hydrogels via enzymatic crosslinking with HRP and H_2_O_2_ with encapsulated betamethasone to increase therapeutic efficacy in the treatment of inflammation in patients with rheumatoid arthritis.

**Figure 1:**
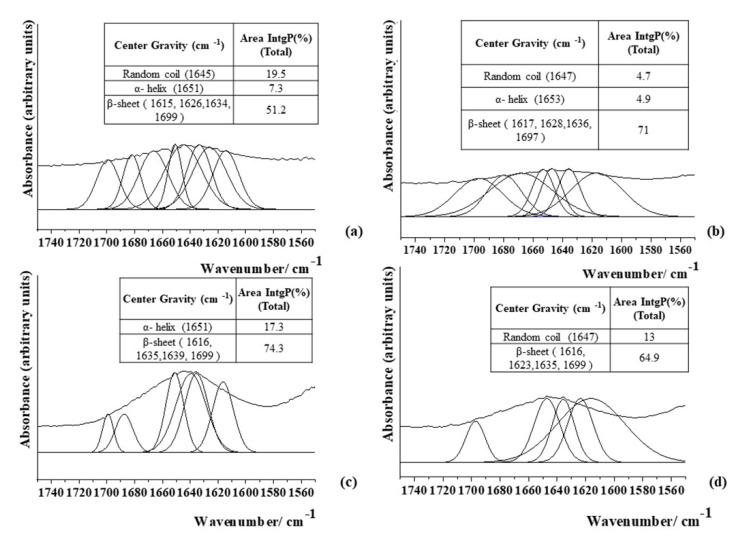
FTIR spectra of secondary structure of the 2% SF solution alone (**a**) and after producing Ty–GG/SF hydrogels via HRP and H2O2, C1 (**b**), C2 (**c**) and C3 (**d**).

Initially, a 1% (*w*/*v*) Ty–GG solution was mixed with a 2% (*w*/*v*) of SF solution to produce Ty–GG/SF hydrogels, followed by physicochemical characterization by FTIR. FTIR methodology was followed to predict the secondary structure of SF in hydrogels. The deconvolution method was made to quantitatively estimate the conformational ratio in the amide I region. The IR spectral region of amine I (1700–1600 cm^−1^) absorption had already been frequently used for the analysis of different secondary structures of SF. The intensity ratios of the β-sheet structure over the α-helix and random coils were calculated to study the relationship between the crystalline and amorphous phases in the SF [30]. The conformational transitions and crystalline content of SF highly influence its physiochemical properties, affecting its biological properties and applications. The secondary structure (Silk II) has an important role in its physicochemical properties and supports its mechanical stabilities [31]. Figure 1 shows the secondary structure of SF 2% (*w*/*v*) alone and after mixing with Ty–GG solution, HRP and H_2_O_2_ (C1, C2 and C3).

The spectrum of 2% (*w*/*v*) SF protein (Figure 1a) revealed an absorption band at 1651 cm^−1^ (7.3%), which is a characteristic peak for an α-helix, and another absorption peak located at 1645 cm^−1^ (19.5%) for a random coil structure (corresponding to Silk I structure). The absorption bands at 1615, 1626, 1634 and 1699 cm^−1^ (51.2%) are features of the β-sheet structure (corresponding to Silk II crystal structure). Quantitative analysis showed—after Ty–GG/SF hydrogels were produced—that the β-sheet structure was the dominant conformation in all conditions (C1 (71%), C2 (74.3%) and C3 (64.9%)), demonstrating that the tested conditions produce more crystalline phases than amorphous; see Figure 1b–d. That is, SF acquired a more stable structure after the formation of Ty–GG/SF hydrogels. The results obtained indicate that the presence of Ty–GG to do enzymatic crosslinking via HRP may have increased the number of β-sheets in the structure of SF.

These results are in line with other studies that showed that modified SF and enzymatic crosslinks were marked by increases of the β-sheet content, that is, improved mechanical properties when compared with SF alone [31,32]. The other studies hypothesized that once SF acquires its mobility within the structure, it is natural to return toward the more stable secondary structure, which is the β-sheet the in the case of SF [33].

**Figure 2:**
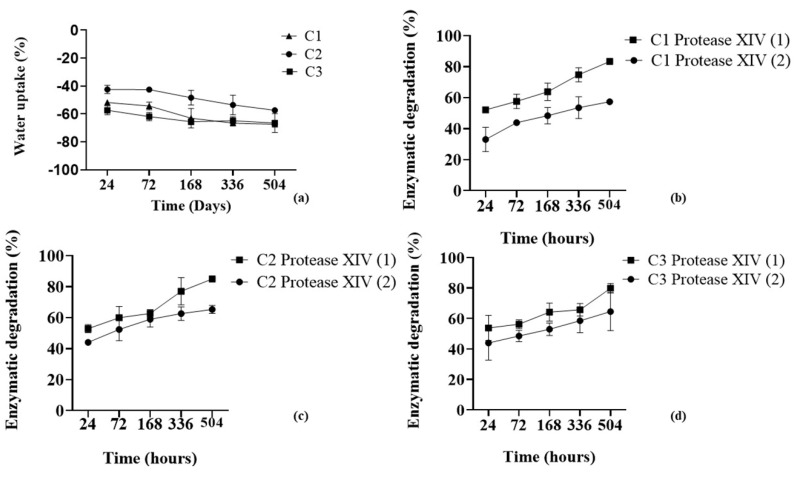
Water uptake (**a**) and enzymatic degradation profiles of C1 (**b**), C2 (**c**) and C3 (**d**) with Protease XIV at 3.3 U mL^−1^ (1) and 1 U mL^−1^ (2) and after 24, 72, 168, 336 and 504 h.

After analyzing the secondary structure of SF, Ty–GG/SF hydrogels (C1, C2, and C3) were evaluated regarding water uptake capacity and enzymatic degradation; see Figure 2.

The swelling kinetics of Ty–GG/SF hydrogels (Figure 2a) were evaluated after they were immersed in PBS solutions (physical crosslinking) for 21 days to maximize crosslinking of the polymeric networks and reach the equilibrium. The water absorption graph showed that all conditions present a negative absorption rate over time, C1 (−57.37% ± 1.12%), C2 (−68.82% ± 5.19%) and C3 (−67.41% ± 0.62%). Regarding the water uptake statistical analysis, there were only significant differences between C1 and C2 (*p* < 0.05) at timepoints 24 and 72 h, by nonparametric Kruskal–Wallis test. The mass swelling ratio has an important role in the mechanical performance, and the combination of chemical and physical leads to the production of hydrogels with high versatility of mechanical properties [34]. The swelling properties are significantly influenced by crosslinking density and ionic osmotic pressure [35]. The chemical crosslink network is highly flexible, allowing the extension of the polymeric network without breaking it. In contrast, the exposure to the physical crosslinking allows the diffusion of monovalent cations within the inner polymeric network and the formation of strong ionic bonds between the chains. These bonds are less flexible, so the capability of the polymer to retain the solvent is decreased by the physical crosslinking. The combination of both crosslinking mechanisms leads to obtaining hydrogels that significantly decrease their mass swelling ratio in PBS [36,37]. The results are in agreement with the studies previously mentioned, which showed that if an external solution is ionically more concentrated than structure, it will promote the entry of ions into the hydrogels, and water molecule expulsion. This allows stabilization and increases the crosslinking of the polymeric chains. [38,39]. Hence, the Ty–GG/SF hydrogels showed a high degree of crosslinking and consequently a good mechanical property. The enzymatic degradation profile was obtained by immersion of Ty–GG/SF hydrogels in PBS solution with sodium azide and diluting the enzyme protease from *Streptomyces griseus* Type XIV, ≥3.5 U mg ^−1^ at 1 and 3.3 U mL^−1^; see Figure 2b–d. The results of the enzymatic degradation showed that condition 1 had a more significant difference in the degree of degradation using the enzyme concentrations 1 U mL^−1^ (57.37% ± 1.2%) and 3 U mL ^−1^ (83.41% ± 0.6%). The same degradation pattern was also found in C2 (66.23% ± 2.12% at 1 U mL ^−1^ and 84.97% ± 0.86% at 3.3 U mL ^−1^) and C3 (66.23% ± 8.82% at 1 U mL ^−1^ and 79.89% ± 2.18% at 3.3 U mL ^−1^). C1, C2 and C3 have similar degradation profiles, but although the differences were not significant, C1 and C3 showed lower degradation profiles over time; that is, those conditions may be slightly more resistant to degradation. Regarding the enzymatic degradation statistical analysis, there were only significant differences between C1, 1 vs. 3.3 U mL ^−1^ (*p* < 0.01) at time point 504 h, C2, 1 vs. 3.3 U mL^−1^ (*p* < 0.05) at time point 24 h, and *p* < 0.01 at timepoint 504 h, by unpaired *t* test. These results may be due to the ratio between the HRP enzyme and H_2_O_2_, which can influence the degree of reticulation of the sample consequently, the degradation profile. Even so, no conditions exposed to the concentration of protease at 1 and 3 U mL^−1^ showed a total degradation profile after 21 days of study. Considering the hydrogels’ degradation profiles, during the time of study, in approximately 1 month the hydrogels could be completely degraded.

Bearing in mind the degradation profiles of Ty–GG/SF hydrogels, it was possible to verify that they are highly resistant to enzymatic degradation when compared to the degradation profiles from other studies, carried out with the same enzyme at different concentrations [13,40].

The gelation time was measured by the inverted tube method. Each condition of Ty–GG/SF hydrogels was produced at room temperature in a tube and monitored by repeat inversion. The gelation time was noted when the solution was no longer flowing upon inversion by the force of gravity, demonstrating the production of a stable gel network. It was possible to observe that all concentrations (C1, C2 and C3) had gelation times around 3–5 min. Gelation time is an essential component for developing the in situ-forming hydrogels, and it is associated with the crosslinking rate of the HRP-catalyzed reaction. HRP and H_2_O_2_ concentrations are crucial criteria to control the crosslinking rate. The gelation time decrease with increasing HRP concentration and vice versa have been studied [41,42]. A slow gelation rate may provoke the heterogeneous distribution of encapsulated entities, so controlled gelation is necessary for efficient local delivery of drugs [43]. Considering the obtained results, it was possible to verify that Ty–GG/SF hydrogels presented a rapid gelation time which may be due to a high degree of enzymatic crosslinking promoted by the HRP enzyme and its H_2_O_2_ substrate.

**Figure 3:**
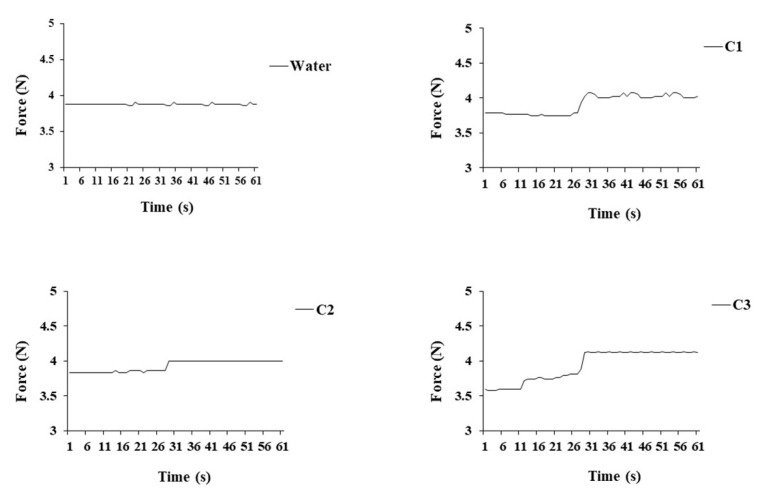
Injectability profiles of Ty–GG/SF hydrogels, C1, C2 and C3 and water (control). There are no significant differences.

Since the hydrogels are intended to be injected into intra-articular cavities, it is crucial to evaluate the injectability profiles of the gels. The injectability, the force required for injection, is a key-product performance parameter to evaluate the performance of the formulation during the injection. The injectability was performed in all conditions, C1, C2 and C3, and as a control water was used. The syringe was filled with the Ty–GG/SF solution, HRP and H_2_O_2_ to produce the hydrogels, and the injection was performed using a rate of 1 mL min^−1^. The obtained injection force profile per condition and that for water (control) are presented in Figure 3.

It was possible to verify that the forces needed to inject all conditions were similar—C1 (3.9 N ± 0.13 N), C2 (3.9 N ± 0.07 N) and C3 (3.9 N ± 0.2 N)—as was the control with the water (3.8 N ± 0.01 N). Firstly, all conditions presented low values of injectability, and at around 30 s, there was an increase in force. This change in values may have been due to the beginning of the crosslinking between molecules, leading to the need for the higher force to inject. However, the value of the force required for each condition remained similar to that of water. This indicated that all conditions have an adequate injectability profile to be used as injectable hydrogels.

**Figure 4:**
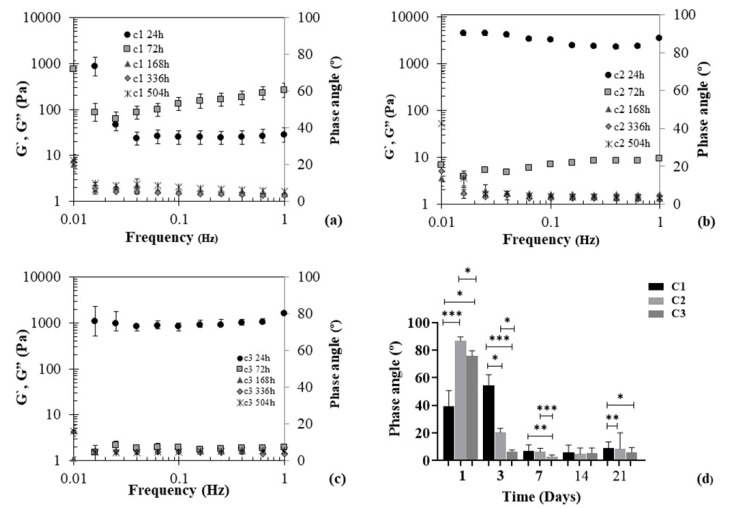
Mechanical spectra of Ty–GG/SF hydrogels C1 (**a**), C2 (**b**) and C3 (**c**) and phase angle values (**d**) at different timepoints (24, 72, 168, 336 and 504 h). G′ (elastic moduli) and G″ (viscous moduli). Significant differences *** *p* < 0.001, ** *p* < 0.01 and * *p* < 0.05, by nonparametric Kruskal–Wallis test. There are not significant differences.

To evaluate the mechanical properties of Ty–GG/SF hydrogels, they were produced, via enzymatic crosslinking, and immersed in PBS, physical crosslinking, for 21 days. At each time point (24, 72, 168, 336, and 504 h), they were removed and evaluated through the oscillatory rheology experiment at 37 °C. The oscillatory shear flow was used extensively to characterize the viscoelastic capacities of materials and to relate them to the polymer structures. The small-amplitude oscillatory shear determines storage modulus (G′) and loss modulus (G″) in terms of stress amplitude and lag between the stress and strain. The phase and amplitude of the relevant portion of the resulting spectra of the stress and strain are used to calculate the storage modulus (G′), loss modulus (G″) and phase angle (δ) for the hydrogel. The phase angle (0 ≤ δ ≤ 90°) is a representative physical property that measures the material viscoelasticity and G′, G″ and δ are determined by equation ((*t*) = **ε*o* sin *ωt*), *εo* (amplitudes of the sinusoidal strain curves) [44,45]. A phase angle of 0° is characteristic of elastic solids; on the other hand, 90° is characteristic of viscous liquids, and between 0° and 90° represents the viscoelastic materials [46]. The mechanical properties of the Ty–GG/SF hydrogels are shown in Figure 4.

The mechanical proprieties resulting from conditions 1 and 2 of Ty–GG/SF hydrogels showed that at time points 24 and 72 h the G″ > G′, and consequently there was a high phase angle, that is, a viscous component higher than the solid. The same pattern was verified in condition 3 at time point 24 h, however, from the third day to last time point G′ > G″, meaning that the solid component was higher than the viscous. The same profile can also be seen in conditions 1 and 2, where there was a higher solid than viscous component from the third day; see Figure 4d. Regarding these results, it was possible to verify that Ty–GG/SF hydrogels had a viscoelastic behavior with a higher elastic component. The cartilage presents viscoelastic mechanical behavior and Ty–GG/SF hydrogels had a mechanical profile similar to the tissue where they will be injected (intra-articular injection); consequently, this material demonstrated a good mechanical capacity for the proposed objective [47]. This equipment could also be used to measure the exact time of gelation by finding the gel point, where the G′ and G″ curves cross. However, the reaction is swift, and the samples quickly acquire the gel character.

Traditional drug delivery approaches present some side effects, such as systemic toxicity and repeating doses. Betamethasone suspensions can be used for intraarticular injection in the inflamed joint. Normal intraarticular doses of betamethasone suspensions vary with the size of the joint, from 1.5 to 12 mg [48]. In the present work, we used 5 mg mL^−1^ of betamethasone since it was the average value found in the literature to have anti-inflammatory activity [23]. Betamethasone has a short half-life of about 36–54 h [49], which means that the drug must be injected more frequently to treat inflammation in the patient. However, hydrogels are suitable drug delivery vehicles to minimize these effects and optimize therapeutic benefits from the drug. The hydrogels have tunable physical properties that confer great controlled drug release and drug protection from degradation. Thus, hydrogels emerged as a very efficient drug delivery system [50,51].

**Figure 5:**
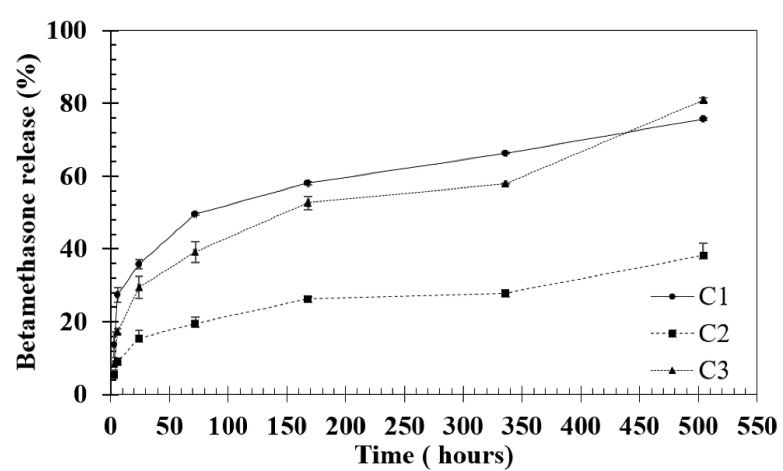
Percentage of betamethasone release from Ty–GG/SF hydrogels (C1, C2 and C3) for 1, 3, 24, 72, 168, 336 and 504 h.

Drug release results showed that after 21 days, C1 presented a betamethasone release profile of 75% ± 0.3% (approximately 3.75 mg mL^−1^), C3 a rate of 80% ± 0.5% (approximately 4 mg mL^−1^) and C2 a rate of betamethasone release of 38% ± 3.3% (approximately 1.9 mg mL^−1^) (Figure 5); that is, no concentration reached the maximum release after 21 days. Significant differences were found at time points 1h and 3 h between C1 and C2 *p* < 0.05, at time point 72h between C1 and C2 *p* < 0.05, at time point 168 between C2 and C3 *p* < 0.05 and at time point 336 h between C1 and C2 * *p* < 0.05, by nonparametric Kruskal–Wallis test. Considering these results, it was possible to state that all conditions had a controlled release profile of betamethasone, which allows prolonged treatment and less frequent drug administration to the patient. Furthermore, this approach can help reduce the toxic effects of drugs. Thus, Ty–GG/SF hydrogels presented the great potential to be used as a betamethasone delivery system.

### 3.2. In Vitro Studies

**Figure 6:**
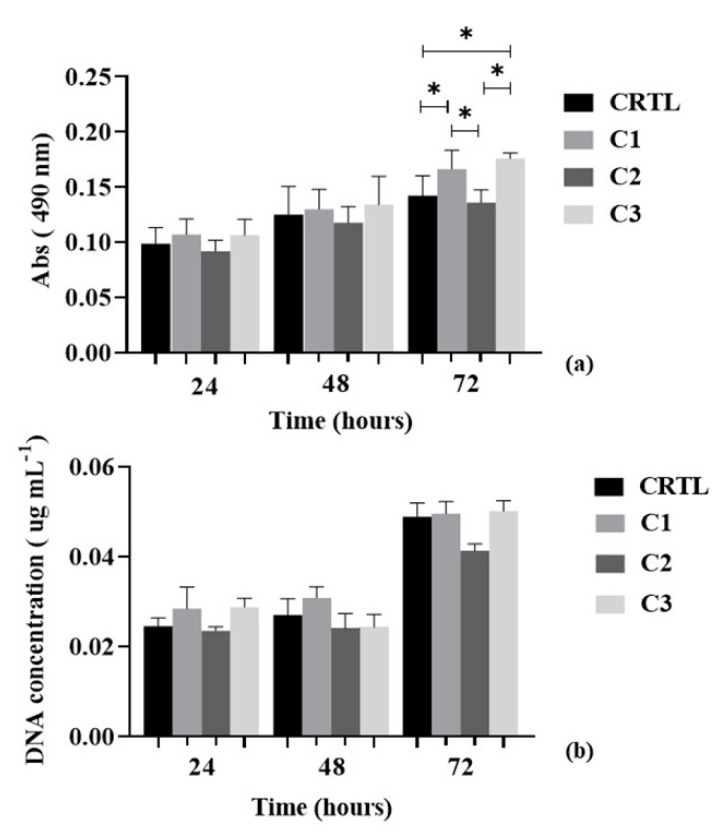
MTT assay (**a**) and DNA quantification (**b**) of chondrogenic primary cells with control (cells with DMEM-12 medium) and different Ty–GG hydrogels conditions (C1, C2 and C3) for 24, 48 and 72 h. Significant differences * *p* < 0.05. Nonparametric Kruskal–Wallis test (MTT assay) and Ordinary one-way ANOVA (DNA quantification).

The MTT assay and DNA quantification were performed in order to assess the metabolic activity and cell proliferation of chondrogenic primary cells with different conditions of Ty–GG/SF hydrogels (C1, C2 and C3) for 72 h; see Figure 6.

At time points 24 h and 48 h, chondrogenic primary cells showed an increase of metabolic activity and we did not observe significant differences between any conditions (*p* > 0.05). At 72 h, there continued to occur an increase in metabolic activity, but we found significant differences between control and C1 (* *p* < 0.05), control and C3 (* *p* < 0.05), C1 and C2 (* *p* < 0.05) and C2 and C3 (* *p* < 0.05)—Figure 6a.

DNA proliferation was quantified using the cell content based on dsDNA quantification; see Figure 6b. An increase in the cell number was observed in the control and C1 for 48 h, and C2 showed approximately the same values in cell proliferation between 24 and 48 h. However, in C3, there was a decrease in cell proliferation between 24 and 48 h, increasing again at 72 h. At the last time point, all conditions demonstrated an increase in cell proliferation and significant differences were not observed. However, the most evident difference was between the (control, C1 and C3) and C2; despite the increase in the number of cells from 48 to 72 h, cell proliferation was lower.

These results are in agreement with the studies found in the literature where the hydrogels of GG and SF do not produce cytotoxic effects on cells [52,53].

**Figure 7:**
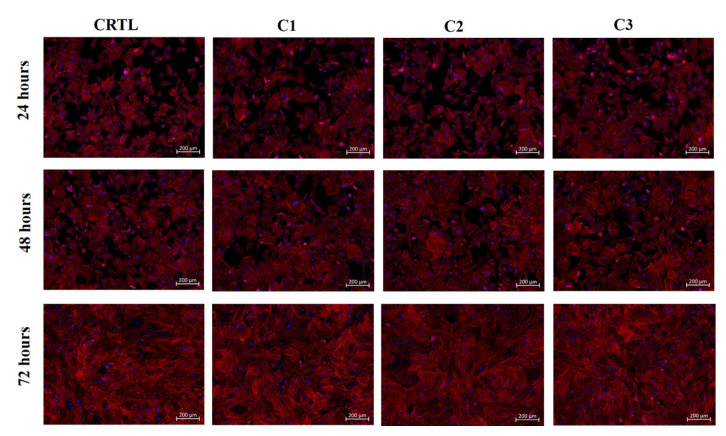
Fluorescence microscopy images of chondrogenic primary cells cultured in the presence of CRTL (RPMI medium) and Ty–GG/SF hydrogels made in different conditions (C1, C2 and C3) for 72 h.

The morphology of chondrogenic primary cells, in contact with Ty–GG hydrogel extract, was analyzed by fluorescence microscopy; see Figure 7.

Chondrogenic primary cells were incubated with C1, C2 and C3 of Ty–GG/SF hydrogels. It was possible to see that all conditions presented the typical morphology of chondrogenic primary cells, blue (nuclei) and red (cytoskeleton), suggesting healthy proliferation and survival when compared with control.

To assess the therapeutic efficacy of Ty–GG/SF hydrogels, the levels of tumor necrosis factor alpha (TNFα) were quantified. In patients with RA, high levels of this cytokine were observed in the synovial fluid, making TNFα an exciting target for RA treatment [5].

Firstly, THP-1 cells were differentiated with PMA and stimulated with LPS to evaluate the capacity of Ty–GG/SF with encapsulated betamethasone to decrease the levels of TNFα present in the RPMI medium. The ability of each of C1 and C3 to decrease TNFα was evaluated because they were the best conditions in metabolic activity and cell proliferation.

After the inflammation was activated, several conditions were evaluated: betamethasone alone (5 mg mL^−1^), and Ty–GG/SF hydrogels (C1 and C3) with and without betamethasone encapsulated (5 mg mL^−1^). Cells differentiated with PMA were used as the negative control and cells stimulated with LPS as the positive control.

**Figure 8:**
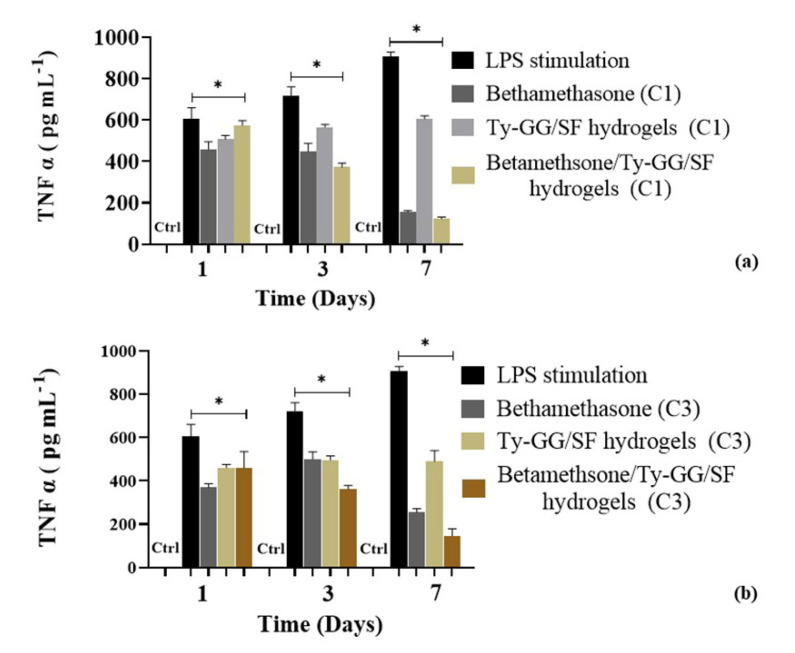
Amount of uncaptured TNFα under different conditions; cells with PMA without LPS stimulation (Ctrl), cells stimulated with LPS, betamethasone (C1), Ty–GG/SF hydrogels (C1) and Ty–GG/SF hydrogels with encapsulated betamethasone (C1) (**a**). Cells with PMA without LPS stimulation (Ctrl), cells stimulated with LPS, betamethasone (C3), Ty–GG/SF hydrogels (C3) and Ty–GG/SF hydrogels with encapsulated betamethasone (C3) (**b**). Significant * *p* < 0.05, nonparametric Kruskal–Wallis test.

Figure 8 shows the amount of TNFα present in RPMI medium in contact with C1 and C3 of Ty–GG/SF hydrogels for 7 days.

The results obtained showed that untreated LPS-stimulated cells showed increasing amounts of TNF over the study period, thereby demonstrating that the stimulation of cells with LPS was achieved. Furthermore, it was possible to verify that the Ty–GG/SF hydrogels (C1 and C3) without encapsulated betamethasone had no effect on decreasing the amount of TNFα. On the other hand, it was observed that the decrease of the TNFα concentration was higher in cells treated with Ty–GG/SF hydrogels, in both conditions (C1 and C3), when compared with betamethasone alone (C1 and C2). This trend continued throughout the study period, and the difference between the therapeutic efficacy of Ty–GG/SF hydrogels with betamethasone encapsulated and the drug alone became more evident at the last point of time, 7 days. This more evident difference may be due to the fact that the hydrogels were being degraded, so there was a higher release of betamethasone at 7 days than at the initial time points, 1 and 3 days.

These results are in agreement with the studies found in the literature where they describe that GG and SF have several unique properties that become favorable for the incorporation and delivery of a range of the therapeutic agents [54,55].

## 4. Conclusions

In this study, drug-loaded, injectable Ty–GG/SF hydrogels were produced to increase therapeutic efficacy in an inflammatory environment. The results obtained are promising enough to envisage a possible application of this product in the inflamed joints of patients with RA.

The Ty–GG/SF hydrogels presented a dominant conformation of β-sheet structure (crystalline phase), thereby indicating that the use of Ty–GG increased the number of β-sheets, that is, a more stable structure when compared with SF alone. The Ty–GG/SF hydrogels showed low water absorption capacity due to the high degree of enzymatic and physical crosslinking. The enzymatic degradation demonstrated that Ty–GG/SF hydrogels exposed to the concentrations of protease of 1 U and 3 U mL^−1^ demonstrated good resistance to degradation—not reaching its maximum after 21 days of study. Furthermore, the hydrogels showed rapid gelation time, good strength and a controlled betamethasone release profile over time, which are appropriate physicochemical characteristics for the final application. The in vitro studies demonstrated that Ty–GG/SF hydrogels did not produce any deleterious effect on cellular metabolic activity, morphology or proliferation. Furthermore, the Ty–GG/SF hydrogels with betamethasone encapsulated revealed greater therapeutic efficacy than the drug alone, and although the difference was not significant, showed that if the study time is increased, the therapeutic effect of hydrogels with betamethasone encapsulated can be even more visible. Therefore, Ty–GG/SF hydrogels can provide an improvement in therapeutic efficacy through controlled drug delivery, thereby overcoming the disadvantages of current strategies for treating rheumatoid arthritis.

## Figures and Tables

**Table 1:** Ty–GG and SF conditions (C1, C2 and C3) with different amounts of HRP and H_2_O_2_ solutions.

	Ty–GG	SF	HRP	H_2_O_2_
C1	83.5 µL	83.5 µL	16.6 µL	10.83 µL
C2	83.5 µL	83.5 µL	18.3 µL	15 µL
C3	83.5 µL	83.5 µL	20 µL	13.3 µL
